# Inexpensive throughfall exclusion experiment for single large trees

**DOI:** 10.1002/aps3.11325

**Published:** 2020-02-14

**Authors:** Benjamin M. Cranston, Breanna F. Powers, Cate Macinnis‐Ng

**Affiliations:** ^1^ School of Biological Sciences The University of Auckland Private Bag 92019 Auckland Mail Center Auckland 1142 New Zealand; ^2^ School of the Environment The University of Auckland Private Bag 92019 Auckland Mail Center Auckland 1142 New Zealand; ^3^ Present address: Department of Biological Sciences Boise State University 1910 University Drive Boise Idaho 83725 USA; ^4^ Te Pūnaha Matatini The University of Auckland Private Bag 92019 Auckland Mail Center Auckland 1142 New Zealand

**Keywords:** drought tolerance, mature forest, plant–water relations, sap flow

## Abstract

**Premise:**

Drought‐induced tree mortality is an emergent threat to forests worldwide, particularly to large trees. Drought‐manipulation experiments involving throughfall exclusion (TFE) tend to focus on large plots that can be expensive to establish and maintain and may be unsuitable for large trees or indigenous forests. We set out to establish a relatively inexpensive TFE method in a natural forest with large trees.

**Methods:**

We designed a novel TFE method and installed it in the Waitākere Range of West Auckland, New Zealand, to study the southern conifer kauri (*Agathis australis*) under long‐term simulated drought. We measured fluxes of water (sap flow) and carbon (stem increment and litterfall) as indicators of drought effects.

**Results:**

Throughfall was cut off to a 22.25‐m^2^ area around individual boles, causing reduced soil moisture and reduced sap flow in droughted trees.

**Discussion:**

Our new TFE method centered on individual, large trees in native forest and is highly customizable to fit other forest and species types. It can be used to assess physiological responses to drought of individual trees independent of stem size.

Under global climate change, drought‐induced tree mortality is rising worldwide (Allen et al., 2010, 2015). Forest water scarcity can have far‐reaching consequences for water and nutrient cycles across a broad range of scales (from single plants to whole ecosystems and from seasonal to multi‐year impacts) (Adams et al., 2010; Anderegg et al., 2013). Impacts include plant water stress, reduced plant water use, reduced productivity and growth, and in extreme conditions, plant mortality (Fisher et al., 2007; Allen et al., 2015). This disturbance leads to ecosystem functional responses, including disruption of forest carbon and water cycles (Anderegg et al., 2013; O'Brien et al., 2017). Methods for detecting, monitoring, and predicting forest drought stress and mortality include remote sensing, forest inventory plots, and mechanistic models (Hartmann et al., 2018). For a detailed understanding of ecophysiological impacts of soil drying, manipulative experiments have been used to simulate drought. For example, indoor common garden experiments allow for manipulation of single or multiple environmental variables (e.g., Creek et al., 2018), but they are only suitable for juvenile trees due to space limitations. Outdoor throughfall exclusion (TFE) plots (e.g., Nepstad, 2002; Fisher et al., 2007), whether in natural or planted systems, divert precipitation, producing dry soil conditions similar to drought.

Beier et al. (2012) reviewed the challenges and limitations of TFE experiments and found that grassland studies are more common than forest studies (46% vs. 30% of studies). Furthermore, the geographical spread of all existing studies around the world is under‐representative of many regions. Of 95 TFE experiments reviewed by Beier et al. (2012), only 4% occurred in the Southern Hemisphere, with no studies in Africa. Mesic sites where vegetation is sensitive to water scarcity were also under‐represented (6% of sites had >1500 mm of rainfall). Many of the tree studies occurred in planted or highly managed forests (not natural systems). Our own survey of the literature found that very few TFE studies incorporate large trees (>50 cm diameter at breast height [dbh]), reflecting the challenges of working with very large plants. Yet we know that large trees can have a disproportionate effect on forest water and carbon budgets (Lutz et al., 2018) and can be particularly sensitive to global change processes (Nepstad et al., 2007; Bennett et al., 2015). By ignoring large trees, we are potentially underestimating the global threat of drought for tree mortality and the consequential impacts on global carbon and water cycles.

Forest‐based TFE experiments range in size from small‐scale single‐species plantations (e.g., in Massachussetts: Borken et al., 2006) to large‐scale multi‐level plots (e.g., in the Amazon: Fisher et al., 2007). Although the former typically allows for extensive sampling and high reproducibility with easy positioning of rain‐out equipment, they suffer from homogeneity of vegetation, insufficient soil microbe content, and artificial environmental variables (Beier et al., 2012). Major limitations presented by the latter experimental set‐up are cost of materials and labor in establishing infrastructure, access to ground site and canopy, and the need to minimize impacts on surrounding forest. A global standardized procedure for conducting drought experiments in “tall stature forests” is described by DroughtNet (https://drought-net.colostate.edu/). This procedure provides detailed designs, measurements, and sampling regimes, and allows for flexibility with certain constraints. However, many forest types, especially dense mature forests, have complicated spatial arrangements of trees and understory plants, which makes installation of established protocols particularly challenging. None of these methods are practical or appropriate for understanding drought impacts in indigenous or old growth forests occurring in inaccessible terrain, which is common in areas of the world, such as New Zealand, that are underrepresented in the literature.

Indigenous forests cover about a quarter of New Zealand's land, but very little is known about effects of drought on these ecosystems. New Zealand kauri (*Agathis australis* (D. Don) Loudon) is one of the largest (by volume) and longest‐lived tree species in the world (Macinnis‐Ng et al., 2016). This ancient southern conifer is culturally significant and highly influential in ecosystem composition, driving species diversity patterns (Wyse et al., 2013); yet *A. australis* is under threat from the soil‐motile *Phytophthora agathidicida* (kauri dieback), a pathogen responsible for widespread death of *A. australis* (Waipara et al., 2013). *Agathis australis* is confined to the north of New Zealand's North Island, where future climate projections predict longer and drier summers (Mullan et al., 2005). We established the *A. australis* TFE experiment to determine the impact of drought on mature *A. australis* trees. Our field site is in a “reasonably virgin state” (Ogden, 1983), with a basal area of 94 m^2^/ha (Wunder et al., 2010). Due to a lack of sufficient funds, an inaccessible site, non‐uniform tree placement, and very large trees (dbh > 50 cm), the DroughtNet protocol was unsuitable for our purposes, leading us to develop the design described in this paper. Given the scarcity of drought response data for large trees and the need for cheaper methods adaptable to a range of ecosystems to address global experimental bias, we have developed a single‐tree TFE method to quantify plant‐scale responses to dry soil. The primary aims for the work were to develop a simulated drought on large trees in a mature forest stand, explore physiological responses, and assess their capacity to withstand drought.

## METHODS

**Figure 1:**
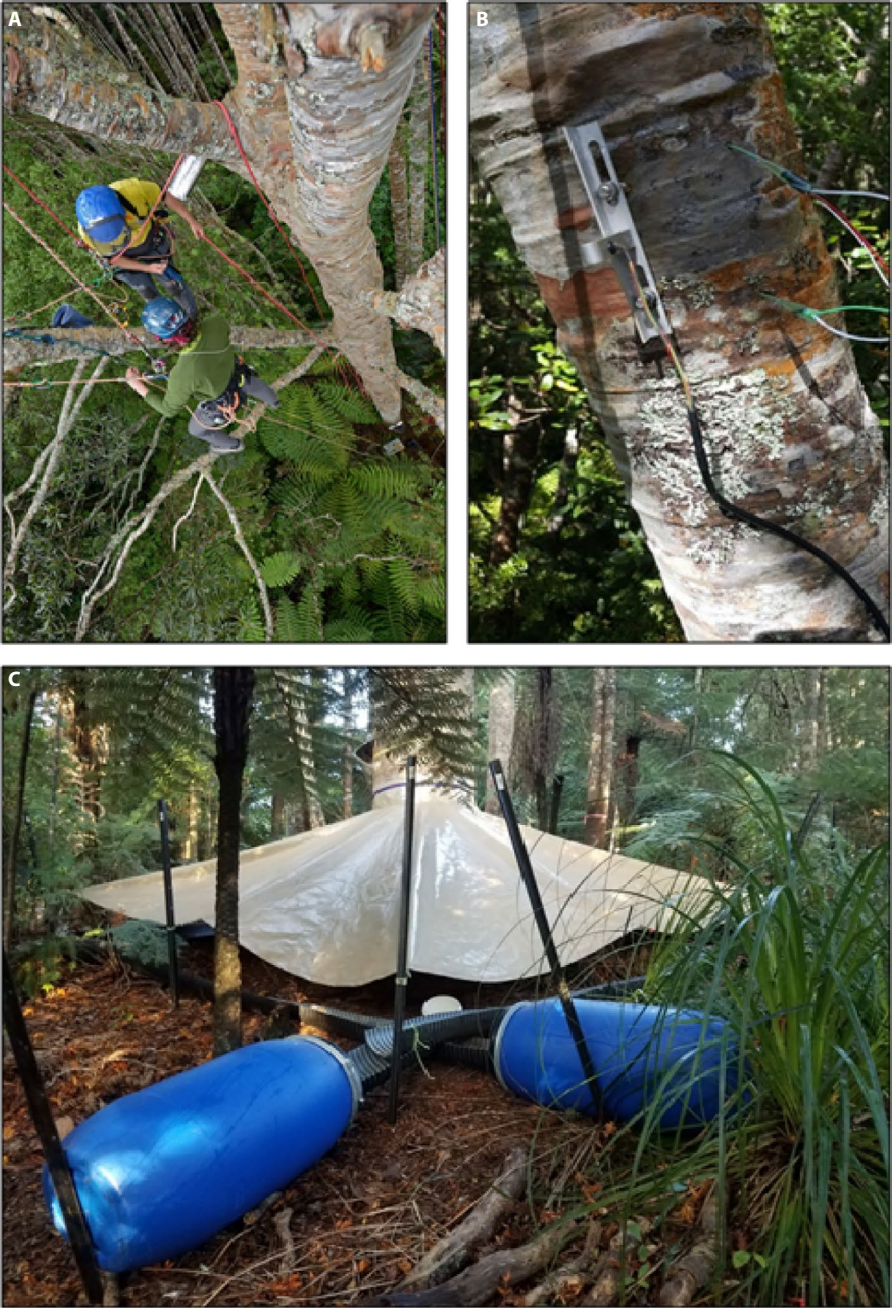
Canopy and base of tree installment stages of the drought experiment. Canopy access for installation of water relations instruments (A), point dendrometer (left) and sap flow sensor (right) installed in a branch (B), and the final set‐up of a droughted tree showing the throughfall exclusion tarpaulin, culverts, and collection basins (C).

A permanent, long‐term (multi‐year) TFE was initialized in mid‐2017 at Huapai Scientific Reserve (36°47.7′S, 174°29.5′E) in western Auckland, New Zealand (Fig. 1). Proximity between study trees, access within the field site, and access to the canopy for undertaking leaf‐scale sampling were considered while choosing the *A. australis* trees. Size/age, slope, adjacent vegetation, and general condition with respect to canopy health were factors for choosing suitable individual trees at the site. Individual *A. australis* trees that were >50 cm dbh were chosen for the TFE (Table 1). To avoid root structure interactions between the six trees (three drought and three control), sample trees were located on two distinct ridges ~200 m apart, with one drought and one control tree on the western ridge and two drought and two control trees on the eastern ridge.

**Table 1:** Kauri drought experiment (KDE) tree details and sensor locations on the tree.

Tree ID	Drought/control	Tree height (m)	Middle sensor height (m)	Branch sensor height (m)	dbh (cm)
KDE1	Control	24.9	8.6	11.8	69.32
KDE2	Drought	27	10.0	14.6	59.48
KDE3	Control	25.1	9.2	13.0	69.91
KDE4	Drought	27.4	10.3	13.0	85.60
KDE5	Drought	25.8	10.9	14.0	67.41
KDE6	Control	25.7	8.8	13.4	54.24

Note

dbh = diameter at breast height.

**Figure 2:**
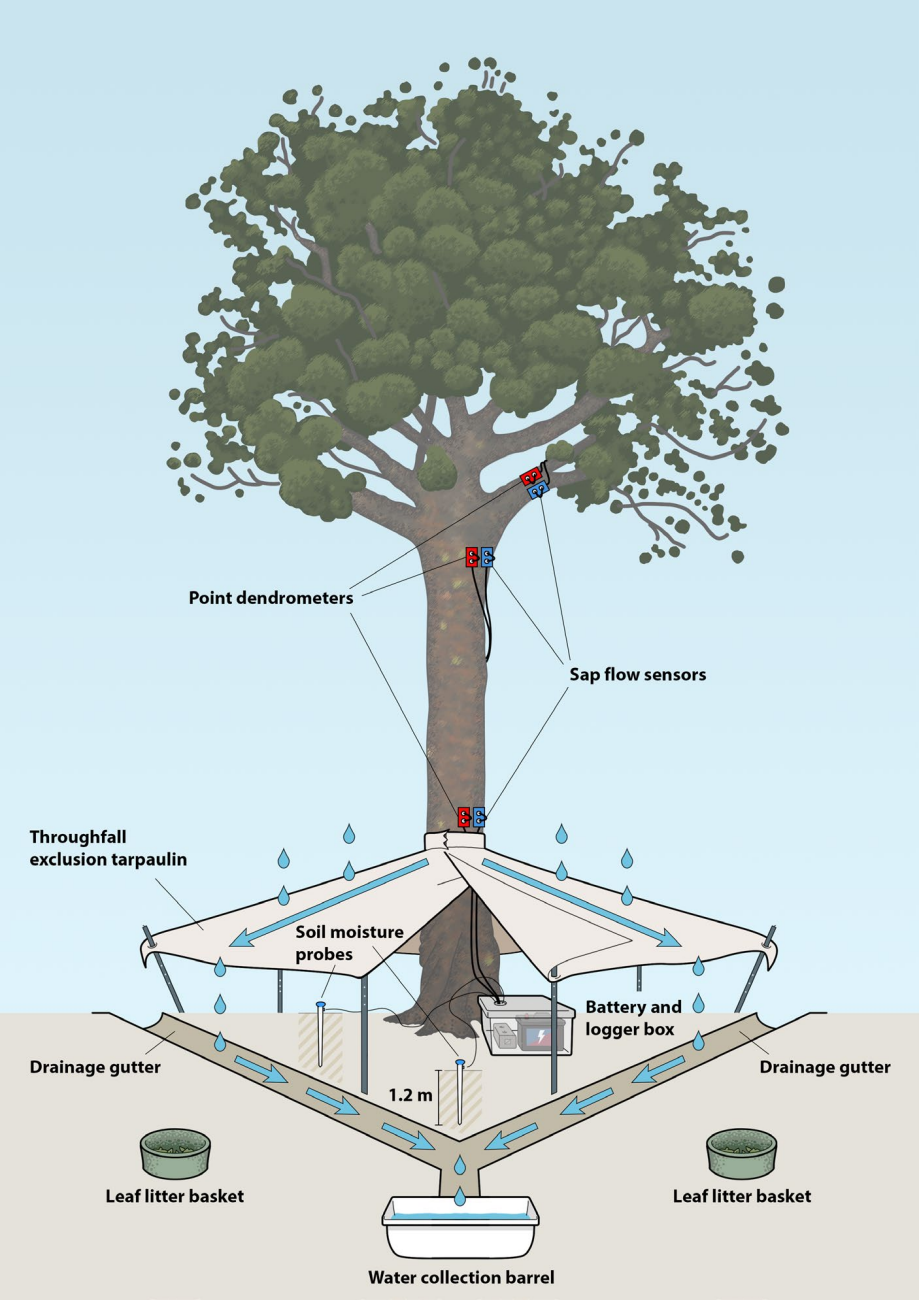
Schematic diagram of drought‐tree experimental apparatus and sensor locations (dendrometer band is not shown but was placed just above the tarpaulin).

To monitor plant responses to drought, we installed a range of manual and automated sensors in the trees and used soil moisture probes to confirm soil moisture drying. We focused on water and carbon cycles as two major forest functions that respond to drought (Anderegg et al., 2013). To quantify biomass (carbon), we used manual and point dendrometers (both show stem growth at different time scales [Wunder et al., 2013; Macinnis‐Ng et al., 2017]), and for water fluxes, we used sap flow sensors (to measure water use [Macinnis‐Ng et al., 2013, 2016]) and point dendrometers (to measure water deficit in the stems [Kaplick et al., 2018]). In each tree, we installed one band dendrometer (UMS GmbH, Munich, Germany) at breast height (1.6 m above the ground on the higher side of sloping ground) and three point dendrometers (Radius dendrometer, Ecomatik, Dachau, Germany). Three modified Granier‐type sap flow sensors (Granier, 1985, with modifications described by Macinnis‐Ng et al., 2016) were installed at breast height, upper stem (below the base of the crown), and low on the first large branch (Fig. 2). Sap flow sensors were installed at 3.5‐cm depth to capture peak radial flow rate (Macinnis‐Ng et al., 2013).

Two soil moisture probes (Drill and Drop Enviroscan 120 cm, Sentek Technologies, Stepney, Australia) were drilled into the ground on the ascending and descending sides of each tree near the trunk (Fig. 2). These probes measure soil moisture and temperature at 10‐cm intervals through the soil profile to 120 cm. With the exception of the band dendrometers, which were recorded manually at three‐week intervals, sensors were connected to data loggers (CR‐1000, Campbell Scientific, Logan, Utah, USA) that recorded at 15‐min intervals. One data logger was located at the base of each tree in a waterproof plastic box. Each system was run using a closed cell battery (12 V, 50 Ah, GS Yuasa, Kyoto, Japan) that was exchanged every three weeks. In addition, two leaf litter baskets (1 m^2^) were placed near the base of each tree, and litter was collected and sorted every three weeks (Macinnis‐Ng and Schwendenmann, 2015). Before the TFE tarpaulins were installed on drought trees, baseline values were taken for two months for all sensors, with the exception of the sap flow and point dendrometer sensors placed in the canopy.

We commissioned a local vendor (Kolorful Kanvas, Christchurch, New Zealand; http://www.kolorfulkanvas.co.nz) specializing in manufacturing waterproof outdoor shelters to produce the custom tarpaulins for the TFE. The material used is commercially known as Canvacon, a woven, polyethylene‐based polyfabric that is UV‐resistant for extended life outdoors. Tarpaulins were 4.5 × 4.5 m draped as a diamond in order to maximize area while avoiding adjacent plants. Each tarpaulin had a hole in the center measuring the circumference of drought tree stems—plus an additional ~4 cm to allow for growth—and ringlets along the perimeter to allow for tying down. Around the stem hole, a ~20‐cm‐tall “collar” runs from the plane of the tarpaulin up the stem in order to deflect stemflow onto the exclusion plane (Fig. 2). Each collar was secured in place with polypropylene webbing and a ratchet that was sufficiently tight to hold the collar in place but not so tight as to constrict the stem, and then wrapped extensively to the stem with shrink wrap for additional waterproofing. The top of the collar needed to allow room above for the breast‐height sensors and dendrometer band on each tree, which limited the height that the edges of the tarpaulin could be elevated above the ground. At the stem, each tarpaulin was attached at a height of 150 cm and, because of complicated topography, the height of the edge of the tarpaulins was between 20 and 60 cm above the ground to allow water to drain into the drainage gutters.

In our case, all three drought trees were on at least a slight incline so gravity aided diversion of captured throughfall. The tarpaulins were pulled taut over the exclusion zone and draped in a diamond pattern with the upper and lower vertices pointed upslope and downslope, respectively. A steel fence post was positioned at each corner of the tarpaulin and tied with a guy‐line to hold the TFE cover in place. Additional guy‐lines were dispersed along the edges of each tarpaulin to hold it firmly in place. Two semi‐flexible high‐density polyethylene (HDPE) drainage gutters (inner diameter of 250 mm) were laid so that each gutter had a 90‐degree bend and covered two edges each of the perimeter of the tarpaulin. Those were individually fed into collection drums (160 L, HDPE) with a semi‐circular cut made in the lid at the bottom vertex. The volume can be collected after small rain events to quantify exclusion efficiency. We decided against trenching as a precaution to prevent the spread of kauri dieback. A full breakdown of costs is outlined in Table 2.

**Table 2:** Approximate costs of equipment used in our experimental set‐up. The first four items are for the TFE experiment and the remaining items are for measuring plant responses to drought.

Supplies	Approximate cost for each unit^a^^,^ b	Approximate USD equivalent (2019)	Total cost of essential items^c^	Comments
Custom‐made tarpaulin	$500	$320	$960	Three tarpaulins in current experiment
Drain pipe for gutters	$20 per m	$15 per m	$450	Could be replaced with cheaper materials such as a plastic‐lined trench. We used 10 m of 250‐mm diameter Maxidrain pipe (cut in half lengthwise) for each tree (30 m in total for three trees).
Water collection drums	$20	$15	$90	Two drums for each drought tree
Items for holding tarpaulins in place	$45	$28	$85	Guy‐lines, stakes, pegs, webbing and ratchet, plastic shrink wrap
Subtotal			$1585	
Logger	$3500	$2240	$13,440	We used Campbell Scientific CR1000 Loggers (one per tree, six in total), but cheaper loggers such as Raspberry Pi or handmade loggers may be substituted to save money.
Batteries (12 V, 50 Ah)	$210	$130	$1560	Two batteries for each tree to allow for rotation
Battery/logger box	$80	$50	$300	
Sap flow sensor	$30 for parts $140 for labor	$110	$1980	Handmade, but these can be commercially purchased for about US$770
Point dendrometer	$790	$500	$9000	Handmade options are available.
Band dendrometer	$25	$15	$75	
Soil moisture probes	$2100	$1340	$16,080	Requires a power drill for installation. Cheaper options are available (e.g., Campbell Scientific CS616 probes are ~US$200 each), reducing overall cost to US$7200 if six sensors were installed per tree.
Audio cable	$800 per 1000 ft	$510	$510	
Litter baskets and stakes	$30	$20	$120	
Climbing labor for sensor installation	$3000	$1900	$1900	Installation took place over 2 d with professional arborists due to health and safety reasons.
Subtotal			$45,055 ($12,335)	The number in parentheses represents costs using handmade or commercially available cheaper options.
Total			$46,550 ($13,830)	The number in parentheses represents costs using handmade or commercially available cheaper options.

a Items such as scientific loggers and point dendrometers are more expensive in remote countries like New Zealand that rely on local suppliers, have lower demand for products due to a small population size, and have increased freight charges that increase final costs.

b Costs are before tax (New Zealand dollars).

c Costs are for three drought trees and three control trees (U.S. dollars).

Meteorological data were collected at a station located approximately 1 km from the experimental site. Data were recorded in 30‐min data intervals for air temperature (°C) and relative humidity (%) with a Vaisala HUMICAP probe (HMP155, Vaisala, Vantaa, Finland) protected in a radiation shield, photosynthetically active radiation (PAR, μmol m^−2^ s^−1^) with a LI‐COR quantum light sensor (Li‐190, LI‐COR Biosciences, Lincoln, Nebraska, USA), rainfall by a tipping bucket rain gauge (RIM8020, MEA, Magill, South Australia, Australia), and wind speed and direction with a cup anemometer and vane, respectively (WMS301, MEA). Apart from the tipping bucket, sensors were mounted on a weather station tripod (MEA) and logged onsite (CR10X, Campbell Scientific, Logan, Utah, USA). Additional cylinder rain gauges were kept at the meteorological station and in the open close to the study site to compare precipitation variance. There was minimal difference between the manually measured rainfall at the meteorological station and closer to the forest.

For data presentation, meteorological data were compiled. Vapor pressure deficit (VPD) values were derived from temperature and relative humidity. Soil moisture was averaged for drought and control trees (six sensors for drought and control each as there were two per tree). We only show a selection (depths for 20 cm and 40 cm) of soil moisture for clarity. For each tree, sap flow values from the base of the stem and the base of the canopy were averaged. These were then averaged to produce representative sap flows for drought and control trees. For point dendrometers, we followed a similar process as sap flow to produce representative radial change for drought and control trees.

## RESULTS

**Figure 3:**
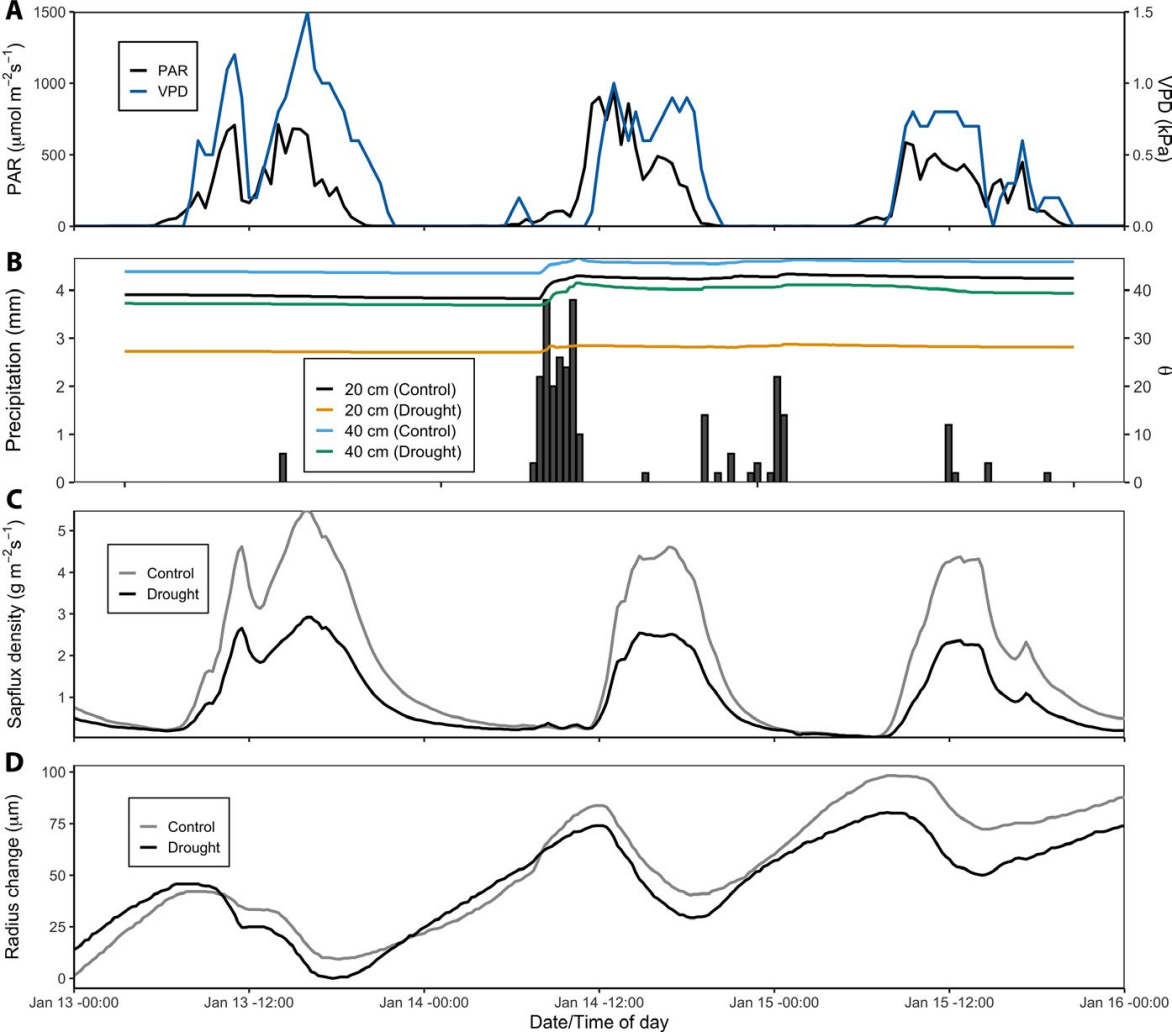
Environmental and meteorological conditions for the period 13 January 2019 (start time: 00:00) through 16 January 2019 (end time: 00:00), a representative three‐day period of our ongoing data set. Vapor pressure deficit (blue line, kPa) and photosynthetically active radiation (PAR) (black line, μmol m^−2^ s^−1^) (A), rainfall (filled bars, mm) and volumetric soil moisture (θ, %) at 20 (black for control and yellow for drought) and 40 cm (green for control and pink for drought) belowground (B), mean sap flux density (g m^−3^ s^−1^) for control and drought trees (C), and mean radial stem change (μm) for control and drought trees (D). Soil moisture values are means of probes among group type (drought: *n* = 6 and control: *n* = 6), sap flux only shows the base sensors per group type (*n* = 3), and radial stem data sets are mean values for all three dendrometers on each tree per group type (*n* = 9). The meteorological data (PAR, VPD, and rainfall) were collected at our meteorological station 1 km from the forest site.

The tarpaulins were installed in December 2018. Here, we present three days of summer data (from January 2019) after approximately one year of imposed drought. There is compelling evidence in our preliminary data set that our experimental design induced drought effects (Fig. 3). There is a clear difference between the soil moisture patterns of drought and control trees (Fig. 3B). Curves for drought trees are consistently lower at both 20‐cm and 40‐cm depths. Deeper soil moisture values showed similar trends for drought and control trees. A sizeable rain event before noon on 14 January appeared to immediately increase the moisture content for both drought and control trees, but the drought trees experienced a more muted response (Fig. 3B). We found lower sap flux peaks and less refilling of stem water in our drought trees for the three days of representative data. Sample data of meteorological indicators, VPD and PAR (Fig. 3A), rainfall (Fig. 3B), volumetric soil moisture at 20 and 40 cm (θ, Fig. 3B), sap flux density (Fig. 3C), and stem radius expansion (Fig. 3D) over a three‐day period during austral summer (growing season for *A. australis*) show some clearly visible differences between drought and control trees.

The sap flux patterns present convincing evidence that VPD and PAR are drivers of transpiration. The prominent dips in sap flux for both drought and control trees at around midday on 13 January coincided with temporary declines in VPD and PAR, and the diminished peaks in sap flux over the subsequent two days were to be expected after the rain event in the morning of 14 January. Comparing sap flux between drought and control trees, the lower flux rates by the drought trees (seen as lower peaks and a smaller area under the curve) over the three‐day period indicate less soil water uptake (Fig. 3C). Diurnal sap flow curves have a characteristic tail that continues after the sun has gone down, when nighttime refilling is occurring. Our control trees underwent more refilling as evidenced by the fact that their evening sap flux densities were higher than those of the drought trees. The radial oscillation of the point dendrometers also showed that the drought trees refilled less, on average, after peak sap flow hours (midday), likely due to less soil water availability (Fig. 3D). We expected litterfall to increase in drought trees, but there was no significant difference between litterfall biomass for drought and control trees to date (data not shown).

## DISCUSSION

Our individual tree‐centered design for installation in natural forests is inexpensive and effective. Preliminary data show differences in soil moisture between trees at depths of 20 and 40 cm, with and without drought tarpaulins, and this was shown to cause differences in sap flow patterns and daily radial expansion of stems (Fig. 3). Our method is especially useful for large trees that may not fit into a plot design of existing TFE methods. Our experimental trees range in size from 54 to 85 cm dbh (Table 1), while trees at our site have dbh values of up to 200 cm (data not shown). The skirt design is flexible as the area of the tarp can be increased to cover a larger portion of root distribution and the center hole that accommodates the stem can also be varied when the tarpaulins are custom made. The tarpaulins are also highly portable and can be carried into remote locations (packed dimensions are approximately 100 × 50 × 80 cm, with a weight of less than 10 kg).

Our method captures responses of carbon and water cycles in forests with large dominant species. We have traded off the capacity to explore community impacts for the ability to measure impacts on individual large trees because large trees are under‐represented in the literature but are highly vulnerable to global change processes (Bennett et al., 2015). Furthermore, studying large trees is a good starting place for understanding ecosystem responses to climate extremes because dominant species strongly influence ecosystem functions, such as fluxes of carbon and water (Felton and Smith, 2017). Although we acknowledge the importance of studying whole community responses, this can be impractical or cost‐prohibitive in forests with very large trees.

Like all TFE methods, our approach has some limitations. TFE experiments are much more challenging in forest systems than in grasslands (Beier et al., 2012), and many of the difficulties and artifacts of plot‐based TFE experiments also apply here (e.g., low replication, potential lateral flow, lack of atmospheric feedback, shading and nutritional artifacts). One potential improvement with our method is that uneven water distribution is not an issue with a tree‐centered approach (Beier et al., 2012); however, it is important to be aware of other artifacts specific to our experimental design. Lateral ground flow can be diverted with effective trenching around the root system, but we chose not to install the trenches both because of kauri dieback present in nearby forests (Waipara et al., 2013) and the ecological and cultural sensitivity of the study species. We were advised that disturbing the soil posed too great a risk to the trees, so we prioritized maintaining the good health of the site. The soil at our site is a heavy clay, meaning that lateral movement of water is minimal, but we would recommend trenching in study locations where it does not pose a major ecological threat.

Another consideration is that the tarpaulins likely cut off stem flow to droughted trees entirely. Stem flow can be an important source of nutrients for plants (Macinnis‐Ng et al., 2012), so our method may confound nutrient and water availability. However, because nutrient flow is driven by the hydrological cycle, reduced rainfall and reduced nutrient availability will both be characteristic of future droughts.

Drought responses often occur at the leaf scale; therefore, in addition to the whole tree measurements reported here, we are also carrying out seasonal canopy sampling. For *A. australis*, VPD and PAR are strong drivers of sap flow rates (Macinnis‐Ng et al., 2016), so measuring leaf‐scale responses will provide further insight into underlying physiological mechanisms that drive changes in whole plant water and carbon fluxes. We are recording leaf gas exchange (photosynthetic rates and stomatal conductance), water potentials of terminal branches, tissue relative water content, turgor loss point, whole‐plant hydraulic conductance, and non‐structural carbohydrates in leaves. Other indicators that could be later added to the measurement regime include belowground processes (e.g., soil CO_2_ efflux, litter decomposition, microbial activity, fine root growth), further measures of plant responses and traits (e.g., dynamics of non‐structural carbohydrates of different tissues, leaf‐scale growth, plant hormones, other metabolic processes, tree‐ring characteristics, wood anatomy, isotopes of leaves for water use efficiency), and imaging of canopy changes (i.e., LIDAR or laser scanning for 3D visualizations).

The experiment will extend until at least August 2020. By this time, we expect that differences in sap flow and radial change will become more pronounced between drought and control trees. We expect a stem water deficit to develop as the drought progresses (Kaplick et al., 2018). Point dendrometer data will allow detection of incomplete stem refilling overnight and indicate when to re‐water trees to avoid tree mortality. We also expect litterfall to increase from droughted trees, as leaf area reduction is a recorded drought response in *A. australis* (Macinnis‐Ng and Schwendenmann, 2015). Our approach will allow us to quantify the function of these factors (access to deep water stores, stem capacitance, drought deciduousness) that help *A. australis* avoid drought stress.

In conclusion, we have overcome challenges of expense and logistics to produce a method that is suitable for iconic and ecologically significant large trees. Large trees dominate forest water cycles and ecosystem carbon storage (Stephenson et al., 2014; Bennett et al., 2015; Lutz et al., 2018) but are also potentially more vulnerable to drought impacts (Bennett et al., 2015; O'Brien et al., 2017). Application of our method will help address the global decline in large old trees (Lindenmayer and Laurance, 2017) by providing data on drought impacts on large trees.

## Data Availability

The preliminary data set is available on the University of Auckland data repository website (https://auckland.figshare.com/articles/Kauri_drought_experiment_methods_dataset_Cranston_et_al_2020_xlsx/11755155).
